# Supplementary material for: Single-cell transcriptome sequencing for opening the blood-brain barrier through specific mode electroacupuncture stimulation
Source: eLife. 2025 Oct 24;14:RP107938. doi: 10.7554/eLife.107938 (PMC12552013; doi:10.7554/eLife.107938)
Supplement: Supplementary file 11. [file elife-107938-supp11.docx]

**Supplementary File 11. KEGG analysis for MG_cluster1 top genes only (20 smallest P values)**

| **Pathway_ID** | **Pathway_Name** | **S** |
| --- | --- | --- |
| [rno04060](https://www.kegg.jp/entry/rno04060) | Cytokine-cytokine receptor interaction | 2 |
| [rno00590](https://www.kegg.jp/entry/rno00590) | Arachidonic acid metabolism | 1 |
| [rno04611](https://www.kegg.jp/entry/rno04611) | Platelet activation | 1 |
| [rno04550](https://www.kegg.jp/entry/rno04550) | Signaling pathways regulating pluripotency of stem cells | 1 |
| [rno04630](https://www.kegg.jp/entry/rno04630) | JAK-STAT signaling pathway | 1 |
