# Supplementary material for: Single-cell transcriptome sequencing for opening the blood-brain barrier through specific mode electroacupuncture stimulation
Source: eLife. 2025 Oct 24;14:RP107938. doi: 10.7554/eLife.107938 (PMC12552013; doi:10.7554/eLife.107938)
Supplement: Supplementary file 14. [file elife-107938-supp14.docx]

**Supplementary File 14. KEGG analysis for MG_cluster0 top genes only (20 smallest P values)**

| **Pathway_ID** | **Pathway_Name** | **S** |
| --- | --- | --- |
| [rno05202](https://www.kegg.jp/entry/rno05202) | Transcriptional misregulation in cancer | 12 |
| [rno04068](https://www.kegg.jp/entry/rno04068) | FoxO signaling pathway | 8 |
| [rno05323](https://www.kegg.jp/entry/rno05323) | Rheumatoid arthritis | 6 |
| [rno04064](https://www.kegg.jp/entry/rno04064) | NF-kappa B signaling pathway | 6 |
