# Supplementary material for: Single-cell transcriptome sequencing for opening the blood-brain barrier through specific mode electroacupuncture stimulation
Source: eLife. 2025 Oct 24;14:RP107938. doi: 10.7554/eLife.107938 (PMC12552013; doi:10.7554/eLife.107938)
Supplement: Supplementary file 23. [file elife-107938-supp23.docx]

**Supplementary File 23. KEGG analysis for MG_cluster3 top genes only (counts top 20)**

| **Pathway_ID** | **Pathway_Name** | **S** |
| --- | --- | --- |
| [rno04921](https://www.kegg.jp/entry/rno04921) | Oxytocin signaling pathway | 4 |
| [rno05020](https://www.kegg.jp/entry/rno05020) | Prion disease | 3 |
| [rno05030](https://www.kegg.jp/entry/rno05030) | Cocaine addiction | 3 |
| [rno04918](https://www.kegg.jp/entry/rno04918) | Thyroid hormone synthesis | 3 |
